# Supplementary material for: Fumonisin B1 as a Tool to Explore Sphingolipid Roles in Arabidopsis Primary Root Development
Source: Int J Mol Sci. 2022 Oct 26;23(21):12925. doi: 10.3390/ijms232112925 (PMC9654530; doi:10.3390/ijms232112925)
Supplement: Supplementary file 1 [file ijms-23-12925-s001.zip › Supplemental Table S2.pdf]

Supplemental Table S1. List of primer pairs used in this study.

**Usage**	**Primer Name**	**Oligo nucleotide**
**Gene expression**	ERF115-F	GCTCCTCCAACTCAAGATCAAGGG
	ERF115-R	TTTGCGGATCCCGAATTTCAGC
	RAD51-F	CGAGGAAGGATCTCTTGCAG
	RAD51-R	GCACTAGTGAACCCCAGAGG
	BRCA1-F	CCATGTATTTTGCAATGCGTG
	BRCA1-R	TGTGGAGCACCTCGAATCTCT
	PARP2-F	ATGGCGTTCTGCTCCTCTGC
	PARP2-R	GGTGCTGTTTTCCCCACACC
	ACTIN7-F	5'- TCCATGAAACAACTTACAACTCCATCA -3'
	ACTIN7-R	5'- CATCGTACTCACTCTTTGAAATCCACA -3'
