# Supplementary material for: Fumonisin B1 as a Tool to Explore Sphingolipid Roles in Arabidopsis Primary Root Development
Source: Int J Mol Sci. 2022 Oct 26;23(21):12925. doi: 10.3390/ijms232112925 (PMC9654530; doi:10.3390/ijms232112925)
Supplement: Supplementary file 1 [file ijms-23-12925-s001.zip › Supplementary Figures.pdf]

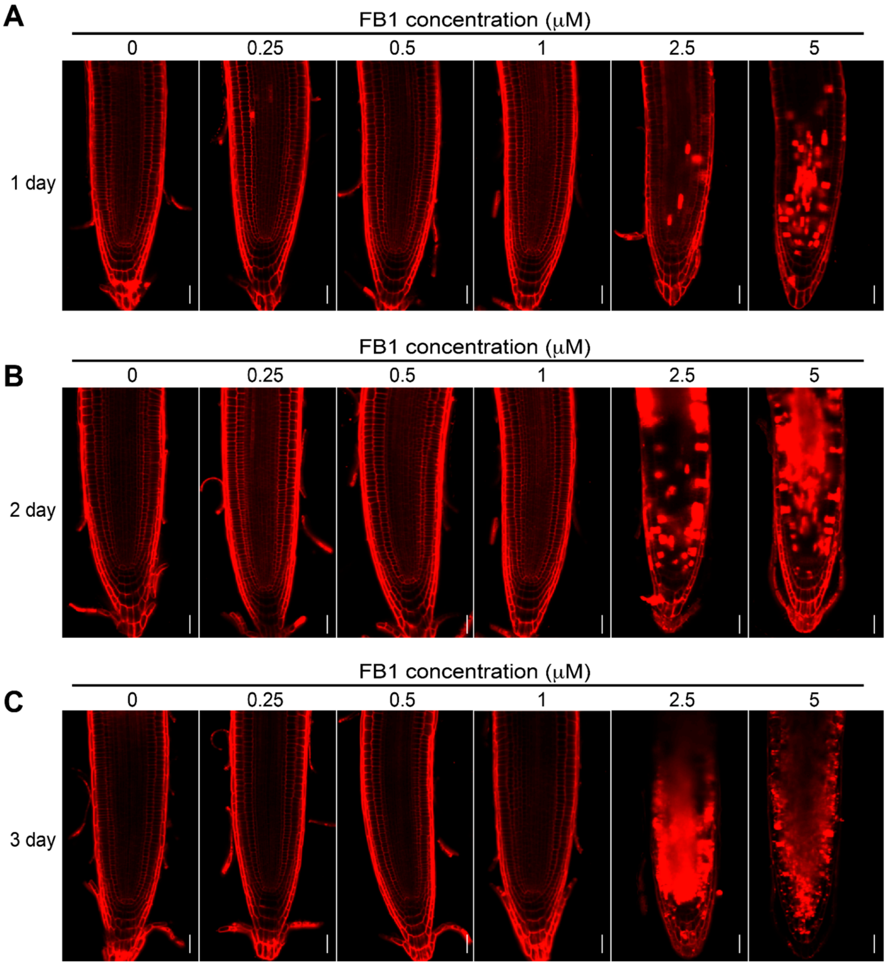

Supplemental Figure S1. FB1 induced cell death in the root apical meristem. (**A**) Confocal images of WT seedlings treated with FB1 at different concentration (0, 0.25, 0.5, 1, 2.5, and 5 μM) for 1 day. (**B**) Confocal images of wild-type seedlings treated with FB1 at different concentration (0, 0.25, 0.5, 1, 2.5, and 5 μM) for 2 days. (**C**) Confocal images of wild-type seedlings treated with FB1 at different concentration (0, 0.25, 0.5, 1, 2.5, and 5 μM) for 3 days. Bars = 50 μm.

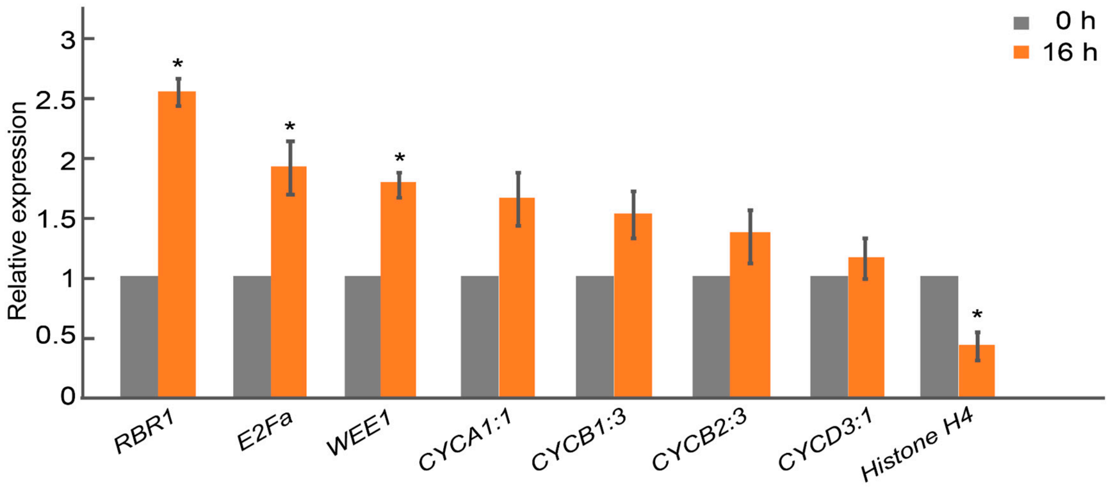

Supplemental Figure S2. relative expression of key cell cycle related genes.

Relative transcript levels of cell cycle related genes in wild-type seedlings treated with 2.5 μM FB1 for 0 or 16 h, as determined by RT-qPCR analysis. Data are means ± SD. Asterisk (*) denotes significant difference relative to seedlings without FB1, as determined by t-test; $P < 0.01$.
